# MicroRNAs Clustered within the 14q32 Locus Are Associated with Endothelial Damage and Microparticle Secretion in Bicuspid Aortic Valve Disease

**DOI:** 10.3389/fphys.2017.00648

**Published:** 2017-09-05

**Authors:** Neus Martínez-Micaelo, Raúl Beltrán-Debón, Gerard Aragonés, Marta Faiges, Josep M. Alegret

**Affiliations:** ^1^Grup de Recerca Cardiovascular, Institut d'Investigació Sanitària Pere Virgili, Universitat Rovira i Virgili Reus, Spain; ^2^Servei de Cardiologia, Hospital Universitari de Sant Joan, Universitat Rovira i Virgili Reus, Spain

**Keywords:** microRNA, bicuspid aortic valve, aortic dilation, circulating endothelial microparticles, bioinformatics, endothelial damage, co-expression network

## Abstract

**Background:** We previously described that PECAM^+^ circulating endothelial microparticles (EMPs) are elevated in bicuspid aortic valve (BAV) disease as a manifestation of endothelial damage. In this study, we hypothesized that this endothelial damage, is functionally related to the secretion of a specific pattern of EMP-associated miRNAs.

**Methods:** We used a bioinformatics approach to correlate the PECAM^+^ EMP levels with the miRNA expression profile in plasma in healthy individuals and BAV patients (*n* = 36). In addition, using the miRNAs that were significantly associated with PECAM^+^ EMP levels, we inferred a miRNA co-expression network using a Gaussian graphical modeling approach to identify highly co-expressed miRNAs or miRNA clusters whose expression could functionally regulate endothelial damage.

**Results:** We identified a co-expression network composed of 131 miRNAs whose circulating expression was significantly associated with PECAM^+^ EMP levels. Using a topological analysis, we found that miR-494 was the most important hub within the co-expression network. Furthermore, through positional gene enrichment analysis, we identified a cluster of 19 highly co-expressed miRNAs, including miR-494, that was located in the 14q32 locus on chromosome 14 (*p* = 1.9 × 10^−7^). We evaluated the putative biological role of this miRNA cluster by determining the biological significance of the genes targeted by the cluster using functional enrichment analysis. We found that this cluster was involved in the regulation of genes with various functions, specifically the “cellular nitrogen compound metabolic process” (*p* = 2.34 × 10^−145^), “immune system process” (*p* = 2.57 × 10^−6^), and “extracellular matrix organization” (*p* = 8.14 × 10^−5^) gene ontology terms and the “TGF-β signaling pathway” KEGG term (*p* = 2.59 × 10^−8^).

**Conclusions:** Using an integrative bioinformatics approach, we identified the circulating miRNA expression profile associated with secreted PECAM^+^ EMPs in BAV disease. Additionally, we identified a highly co-expressed miRNA cluster that could mediate crucial biological processes in BAV disease, including the nitrogen signaling pathway, cellular activation, and the transforming growth factor beta signaling pathway. In conclusion, EMP-associated and co-expressed miRNAs could act as molecular effectors of the intercellular communication carried out by EMPs in response to endothelial damage.

## Introduction

Bicuspid aortic valve (BAV), the most common cardiac congenital malformation (occurs in 1–2% of the population), is associated with valve dysfunction and is a risk factor for aortopathy (Tzemos et al., 2008; Alegret et al., 2013). The progressive dilation of the aorta, if untreated, can lead to fatal consequences such as aortic dissection and/or rupture. The mechanisms that underlie aortic dilatation have been a matter of debate for years (Padang et al., 2013); the proposed causes include anomalous flow in the ascending aorta generated by the anomalous dynamics of BAV (Kim et al., 2012; Bissell et al., 2013) and genetic causes responsible for the anomalous structure of the aortic media (Biner et al., 2009; Pepe et al., 2014).

In a previous study, we reported that circulating endothelial microparticles (EMPs) are elevated in BAV patients and related to aortic dilation (Alegret et al., 2016). Circulating EMPs are a type of extracellular microvesicle (100–1,000 nm) that bud directly from the plasma membranes of endothelial cells upon activation, injury, or apoptosis and that are involved in intercellular communication. Specifically, we identified PECAM^+^ EMPs, which are a kind of EMP that express CD31 and are released in endothelial damage, as those related to BAV disease.

Micro-ribonucleic acids (miRNAs) are endogenously expressed, 19- to 23-nt-long noncoding RNAs that regulate gene expression at the post-transcriptional level, mostly via imperfect base-pairing interactions that occur preferentially within the 3′ untranslated regions (UTRs) of target mRNAs. MiRNA genes are distributed across diverse genomic locations, and although some miRNAs are isolated, ~50% are found in clusters transcribed as polycistronic miRNA transcripts (Mourelatos et al., 2002). MiRNAs are considered potent post-transcriptional regulators because each miRNA has multiple to several 100 target genes; therefore, inhibition of a single miRNA can lead to the activation of multifactorial physiological processes. In addition to functioning intracellularly, miRNAs can be exported or released by cells into the circulating blood in very stable forms. Microparticles are reportedly the major carriers of miRNAs in the blood (Diehl et al., 2012). MiRNA signatures have been proposed as potentials with the potential to improve disease diagnosis and prognosis in clinical practice and have been identified as useful biomarkers for a wide range of cardiovascular diseases. For example, in a previous study, we proposed miR-122, miR-130a, miR-486, and miR-718 as molecular features associated with BAV and aortic dilation (Martínez-Micaelo et al., 2017).

Currently, there are no effective strategies to prevent the progression of BAV disease, including the aortic dilation, and the development of new strategies requires more detailed understanding of the molecular mechanisms associated with BAV and progressive dilation of the aorta. Therefore, in this study, we hypothesized that changes in blood flow in the ascending aorta caused by the bicuspid morphology of the aortic valve would induce endothelial damage, resulting in the secretion of a specific signature of EMP-associated miRNAs. We used a bioinformatics approach to integrate the PECAM^+^ EMP levels with the miRNA expression profile in plasma of healthy individuals and BAV patients. In addition, from the miRNAs that were significantly associated with PECAM^+^ EMP levels, we inferred a miRNA co-expression network using a Gaussian graphical modeling approach.

## Methods

### Study population

The patients included in this study belonged to a cohort of BAV patients who were prospectively included and followed-up in our facilities. Upon enrolment, the participants were informed and prospectively entered into a specific database, underwent a blood draw and provided informed written consent. The samples were stored in our biological samples bank (Biobanc IISPV—HUSJR) until they were needed. A BAV diagnosis was made when two aortic leaflets were clearly visualized, with or without a raphe, in the parasternal short-axis view of a transthoracic echocardiogram (Alegret et al., 2005), on a transesophageal echocardiogram (Alegret et al., 2005), or on a cardiac magnetic resonance image (Gleeson et al., 2008). Explorations were performed or supervised by the same observer (JMA). Our database and biobank also included a group of healthy tricuspid aortic valve (TAV) controls. This study was approved by the Institutional Review Board (the Clinical Ethics Committee) of our institution.

This study was designed in two evaluations in which EMP levels and the miRNA expression profile were determined in two independent cohorts of TAV individuals and BAV patients with or without aortic dilation (*n* = 60). In the first evaluation, plasma from the patients diagnosed with BAV, with or without aortic dilation, and healthy TAV control subjects (*n* = 24) was used to determine the levels of EMPs and the miRNA profile using microarrays. To improve the study's power, for this stage, we selected groups composed of patients with characteristics that were extremely homogeneous (Supplementary Table 1) to exclude possible confounding factors, such as age, sex, or BMI. We included subjects in whom high levels of circulating PECAM^+^ EMPs were expected, the BAV patients, and subjects in whom low levels of PECAM^+^ EMPs were expected, the healthy TAV controls, to study miRNAs whose expression levels could be related to PECAM^+^ EMPs. Patients diagnosed with cardiovascular diseases, Marfan syndrome, aortic stenosis, hypertension, or diabetes mellitus or who were receiving pharmacologic treatment (including statins, ACE/ARII, and/or β-blockers) were excluded. In the second evaluation, the EMP-associated miRNA candidates were validated by RT-qPCR in a new cohort (*n* = 36) of TAV healthy controls and BAV patients with or without aortic dilation (Supplementary Table 2).

### Blood sampling

Blood samples were collected under overnight fasting conditions and were processed within 90 min after collection. The samples were centrifuged at 1,500 g for 15 min to obtain plasma, which was further centrifuged at 4,000 g for 10 min to obtain platelet-poor plasma. The plasmas were stored at −80°C in our biological samples bank (Biobanc IISPV—HUSJR) until they were needed.

### Determination of circulating levels of EMPs

The circulating PECAM^+^ EMP levels were determined in the same cohorts used for the microarray and RT-qPCR validation analyses that were previously phenotyped (Alegret et al., 2016), but for this study, individuals were selected to maximize the power of the miRNA analysis. The levels of EMPs were characterized based on the presence of endothelial-specific surface antigens, the composition of which depends on the cellular origin of the microparticles and the generating process (Jimenez et al., 2003). In our previous study (Alegret et al., 2016), we determined that the presence of CD31 (PECAM) is a marker that can discriminate between microparticles released from endothelial cells subjected to endothelial damage produced by haemodynamic causes due to the anomalous aortic flow associated with BAV and microparticles released by other triggering stimuli, including cell activation or apoptosis, in TAV and BAV patients. The concentration of circulating PECAM^+^ EMPs was determined on an EPICS-XL (Beckman Coulter) flow cytometer at a low rate setting and a 30 s stop time. The Nano Fluorescent Particle Size Standard Kit (Spherotech) was used for instrument standardization, and Flow-Count fluorospheres (Beckman Coulter) were added as an internal calibrator to calculate microparticle amounts.

Plasma EMPs were labeled by incubating 50 μl of platelet-poor plasma with the corresponding antibody, anti-CD31-PE (Beckman Coulter), anti-CD42b-FITC (Beckman Coulter), or anti-CD45-PE (Beckman Coulter), at room temperature in the dark for 20 min as previously described (Ci et al., 2013). Then, 500 μl of PBS was subsequently added, and the EMP levels were determined as previously described (Jimenez et al., 2003; Sutherland et al., 2010; Ci et al., 2013). EMPs were defined as particles >0.1 and < 1 μm in size, and their endothelial origin was identified based on their affinity to specific cell surface antigens, namely, CD31 and CD42b. To evaluate the extent of possible contamination with leukocyte-derived microparticles, the circulating levels of CD31^+^CD45^+^ microparticles were determined in all samples. We found that < 4.5% of CD31^+^ microparticles co-expressed CD45^+^; this result is consistent with previous reports by other authors (Amabile et al., 2005; Pirro et al., 2006). EMP levels were measured by trained technicians who were blind to the clinical status of the patients as well as to the results.

### RNA isolation and preparation of miRNA microarrays

Total RNA was extracted from 250 μl of plasma using TRIzol reagent according to the manufacturer's instructions (Invitrogen) and purified using an RNeasy minikit (Qiagen). To increase RNA recovery, 1 μg of MS2 carrier RNA was added to each plasma sample. The quality of total isolated RNA was determined using the Agilent 2100 Bioanalyzer.

The plasma miRNA expression levels were assessed using Sure Print G3 human 8 × 60 k miRNA microarrays (Agilent Technologies) covering 1,205 human miRNAs (Sanger miRBase release 16). The miRNAs were dephosphorylated and labeled with cyanine 3-cytidine biphosphate including a labeling spike-in solution (Agilent Technologies) to assess labeling efficiency. The samples were hybridized on the arrays with the inclusion of a hybridization spike-in solution (Agilent Technologies) to monitor hybridization efficiency. The arrays were scanned with a G2565CA Microarray Scanner System with SureScan High-Resolution Technology (Agilent Technologies) using Scan Control software. The Feature Extraction 11.5.11 (Agilent Technologies) and GeneSpring 12.6.1. software packages were used for data processing.

### Analysis of miRNA microarray expression data and integrative analysis and gene co-expression network

The data from microarrays (deposited at Gene Expression Omnibus under GEO Series accession number GSE101616) were normalized using the robust multi-array average (RMA) method (Irizarry et al., 2003) implemented in the AgiMicroRna Bioconductor package, and the fold changes in circulating miRNAs were determined using the linear model implemented in the limma Bioconductor package (Ritchie et al., 2015). The Benjamini and Hochberg methods were used to adjust *p*-values for multiple testing and to control the false discovery rate (Benjamini and Hochberg, 1995). The miRNA co-expression network was constructed from the expression profiles of those miRNAs whose expression was significantly associated with PECAM^+^ EMPs (Spearman *p* < 0.05). The co-expression network was inferred using graphical Gaussian models (GGMs) implemented in the R package GeneNet (Schäfer and Strimmer, 2005). Briefly, a partial-correlation matrix was estimated by computing the partial correlation between the expression profiles of each miRNA pair. Bayesian posterior edge probability >0.95 (corresponding to a local false discovery rate of < 5%) was used to determine the significance of the resulting pairwise partial correlations. In the resulting co-expression network, which was visualized using Cytoscape software (Cline et al., 2007), the nodes represent the set of miRNAs that were significantly correlated with PECAM^+^ EMP levels, and the edges link the pairs of miRNAs whose expression was not conditionally independent, defined as the pairwise partial correlation once the common effects of the other miRNAs in the subset were removed (Opgen-Rhein and Strimmer, 2007).

### MiRNA quantification by real-time qRT-PCR

TaqMan microRNA assays (Applied Biosystems) were used to quantify the expression of selected miRNAs. Briefly, reverse transcription was performed using the TaqMan MicroRNA Reverse Transcription Kit (Applied Biosystems) and the miRNA-specific oligonucleotides provided with the TaqMan MicroRNA Assay (Applied Biosystems). The final concentration of total RNA used was 2.5 ng/μL. The reaction was performed at 16°C for 30 min, 42°C for 30 min, and 85°C for 5 min. We used 1.33 μL of the obtained cDNAs in a subsequent quantitative qRT-PCR amplification using the TaqMan Universal PCR master mix (Applied Biosystems, Madrid, Spain) and the associated specific probe provided in the TaqMan® MicroRNA Assay Kit (Applied Biosystems). Specific TaqMan probes were used for each gene: let-7d (hsa-let-7d-5p), let-7g (hsa-let-7g-5p), miR-122 (hsa-mir-122-5p), miR-130a (hsa-mir-130a-3p), miR-337 (hsa-mir-337-5p), miR-409 (hsa-mir-409-3p), miR-486 (hsa-mir-486-5p), miR-494 (hsa-mir-494), and miR-718 (hsa-mir-718). The results were normalized to the expression of the U6 small nuclear RNA (U6 snRNA), which was used as an endogenous control. Amplification was performed in a 7900HT thermocycler (Applied Biosystems) at 95°C for 10 min, followed by 40 cycles of 95°C for 15 s and 60°C for 1 min. The fold change in the miRNA level was calculated by the log 2 scale according to the equation 2^−ΔΔCt^, where ΔCt = Ct miRNA-Ct U6 and ΔΔCt = ΔCt treated samples-ΔCt untreated controls. Negative control reactions, with no RNA, had undetectable quantification cycle values (C_q_).

### Genomic region overrepresentation analysis

The genetic loci visualization of co-expressed miRNAs across chromosomes was performed using PhenoGram (Wolfe et al., 2013). To determine the significance of the overrepresentation of chromosome regions in the generated co-expressed miRNA set, we applied a hypergeometric test.

### Target prediction and functional pathway analysis

Putative miRNA target sites for the miRNA candidates were identified by bioinformatics analysis using miRNA target prediction databases, including DIANA-microT-CDS (Paraskevopoulou et al., 2013), DIANA-TarBase v7.0 (Vlachos et al., 2015a), and TargetScan v6.2 (Agarwal et al., 2015). Functional enrichment analysis of miRNA target genes was conducted using miRPath v3.0 (Vlachos et al., 2015b). Visualization and topological analysis of the network was done using R and Cytoscape software.

### Statistical analysis

Because of the right-skewed distribution of the values, the PECAM^+^ EMP plasma levels underwent a natural logarithmic transformation and were expressed as log-transformed counts per μl (log PECAM^+^ EMPs/μl). EMP levels are presented as the mean ± SEM. Chi-squared tests, or Fisher exact tests when appropriate, were used to compare the frequencies of the categorical variables. The effects of valve morphology and aortic root dilation were assessed using ANOVA. Tukey's test was utilized for pairwise comparisons. *p* < 0.05 were considered significant. The statistical analysis was performed using SPSS software, version 21.0 (IBM, Chicago, IL, USA).

## Results

### Identification of circulating miRNA sets associated with PECAM^+^ EMP levels

To explore the circulating PECAM^+^ EMP-associated miRNAs, we first corroborated our previously reported finding that BAV patients have increased levels of PECAM^+^ EMPs in plasma compared with TAV healthy controls (Supplementary Figure 1A). Furthermore, the results of this analysis also supported the role of PECAM^+^ EMP circulating levels as a biomarker of aortic dilation in BAV disease, as an increased diameter of the aortic root or ascending aorta was associated with higher levels of PECAM^+^ EMPs (Supplementary Figure 1B).

We used a microarray-based screening approach to determine the miRNA expression pattern that could be related to endothelial damage. The expression of 1,205 miRNAs were evaluated in plasma samples from BAV patients and TAV healthy controls, and after the miRNA microarray expression data were processed, 277 miRNAs were identified as expressed in at least 5% of the samples. We prioritized the potential miRNA candidates based on the linear relationship between PECAM^+^ EMP circulating levels and the miRNA expression (Supplementary Figure 1C), and based on this analysis, we found that the expression of 175 miRNAs was significantly associated with the circulating levels of PECAM^+^ EMPs (*p* < 0.05).

### Construction of a miRNA co-expression network based on the EMP-associated miRNAs

Once we identified the 175 miRNAs whose expression patterns in plasma significantly correlated with the levels of PECAM^+^ EMPs, we took into consideration that about half of all described miRNAs are co-expressed in clusters of miRNAs. For this reason, we used a Gaussian graphical model approach to map the simultaneous expression of miRNA pairs into a co-expression network.

**Figure 1:**
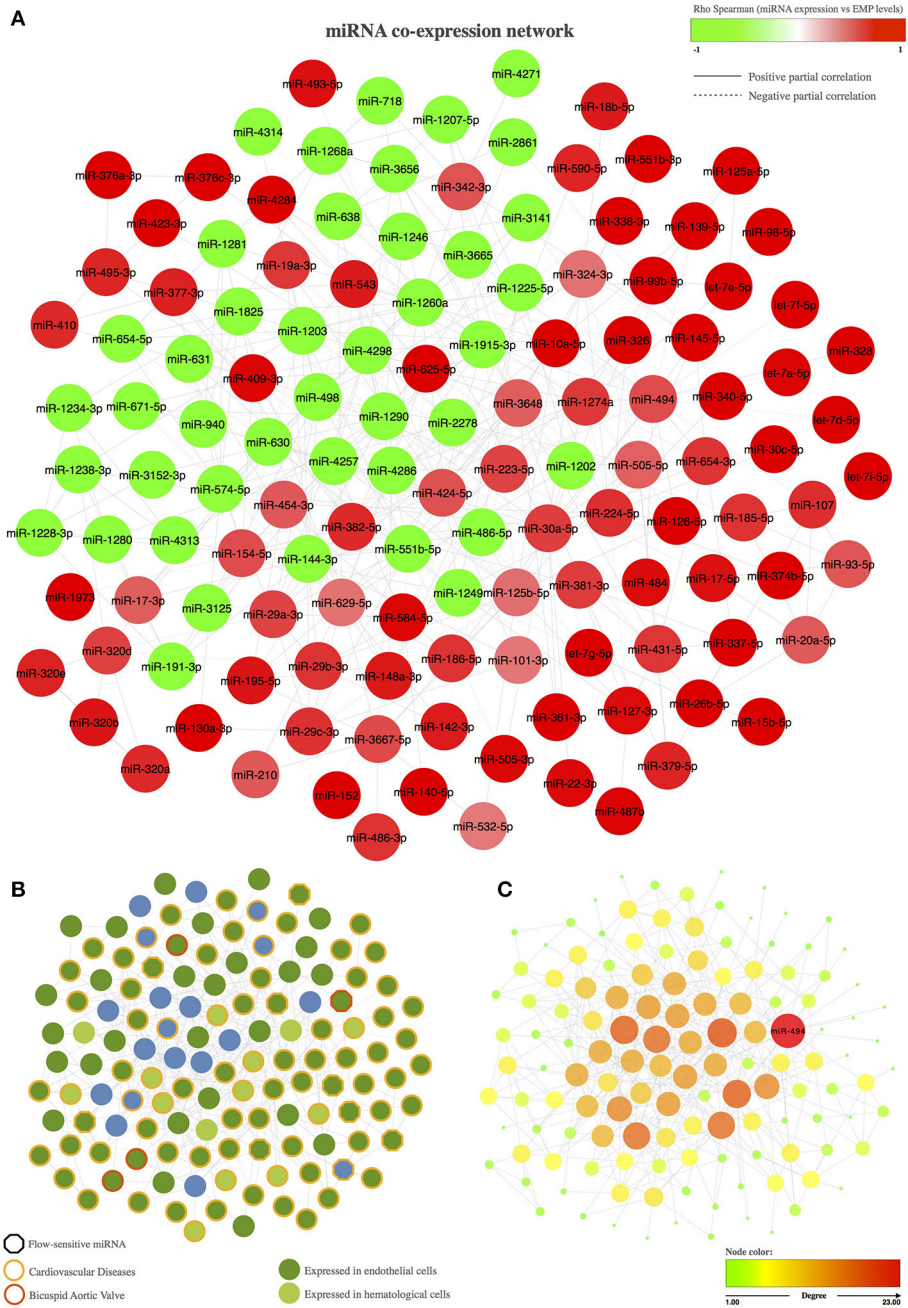
Co-expression network of miRNAs associated with the circulating levels of EMPs. **(A)** Representation of the miRNA co-expression network. The node color represents the Spearman correlation between the expression of the corresponding miRNA and PECAM+ EMP levels; red nodes correspond to miRNAs that are positively correlated with PECAM+ EMPs, and green nodes correspond to miRNAs that are negatively correlated with PECAM+ EMPs. The edge shape is related to the direction of the partial correlation; the continuous lines represent partial positive correlations, and dotted lines refer to partial negative correlation. **(B)** Biological implications of the miRNAs included in the co-expression network based on previous knowledge. The border color of the node represent miRNAs involved in cardiovascular diseases or in bicuspid aortic valve. The hexagon node shape corresponds to flow-sensitive miRNAs. The node color represents miRNA expression in endothelial or hematological cells. **(C)** Topological analysis of the miRNA co-expression network. miR-494 was identified as the master switch based on its role as the most important hub in the co-expression network. The color and the size of the node refer the topological importance of the miRNA within the network. Node positions are conserved between networks.

The resulting miRNA co-expression network is composed of a single connected component formed by 131 miRNAs (FDR < 5%) linked by 391 edges (Figure 1A). We first evaluated the biological implications of these co-expressed miRNAs in terms of intercellular communication in BAV-induced endothelial damage (e.g., whether they are expressed in endothelial cells and whether their expression levels have been previously associated with cardiovascular diseases; Figure 1B). More interestingly, we also focused on miRNAs that were described in previous studies as related to BAV or whose expression might be sensitive to blood flow. We found in the literature that 98 of the 131 co-expressed miRNAs were previously reported as expressed in endothelial cells. Moreover, 88 miRNAs included in the co-expression network had been related to cardiovascular disease. More specifically, modulation of the expression of 4 of them had been associated with BAV, and 9 miRNAs had been described as blood flow sensitive. We also carried out a topological analysis of the network and identified miR-494 as the most important hub within the co-expression network, that is, the miRNA with the largest degree; the highest betweenness, stress, and closeness scores; and the highest radiality coefficient (Figure 1C).

**Figure 2:**
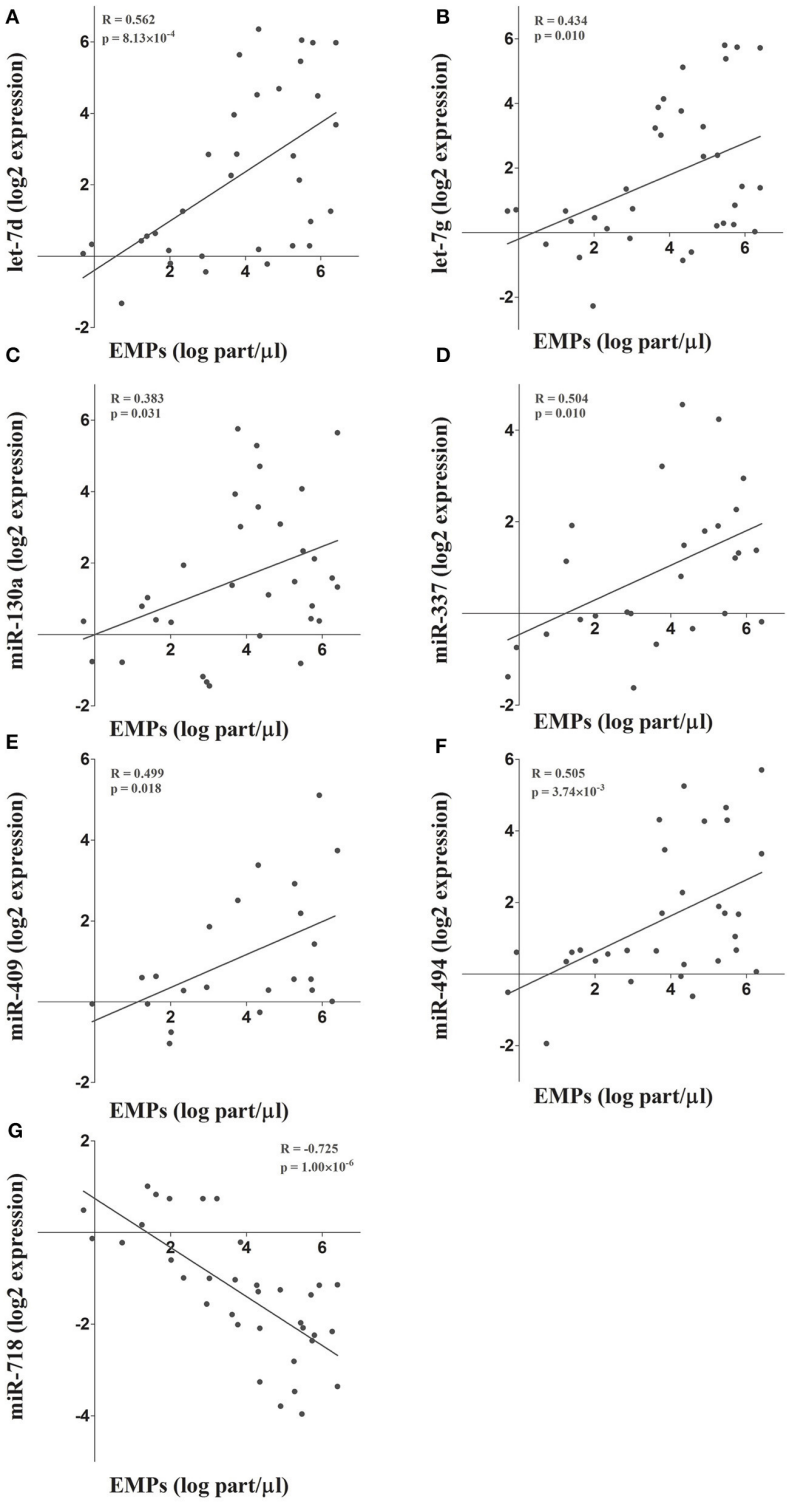
**(A-G)** The microarray results and the integrative analysis were further validated by determining the expression of 7 miRNAs included in the co-expression network. The expression of these miRNAs was determined by RT-qPCR and correlated with circulating PECAM+ EMP levels.

To further validate the results obtained in the microarray analysis and the EMP-miRNA correlations, we determined the expression of 7 of miRNAs included in the miRNA co-expression network (let-7d, let-7g, miR-130a, miR-337, miR-409, miR-494, and miR-718; Figure 2) by RT-qPCR in a new cohort of TAV individuals and BAV patients (*n* = 36). In this way, by correlating the RT-qPCR-determined expression data for each of the selected miRNAs with the circulating levels of PECAM^+^ EMPs, we validated and reaffirmed the sensitivity and power of our bioinformatics approach for the identification and selection of PECAM^+^ EMP-associated miRNA candidates.

### Genomic region enrichment analysis for the miRNA co-expression network

Because co-expressed miRNA pairs tend to reside in close genomic proximity, we performed a positional gene enrichment analysis to identify chromosome regions that were overrepresented among the PECAM^+^ EMPs-associated and co-expressed miRNAs.

**Figure 3:**
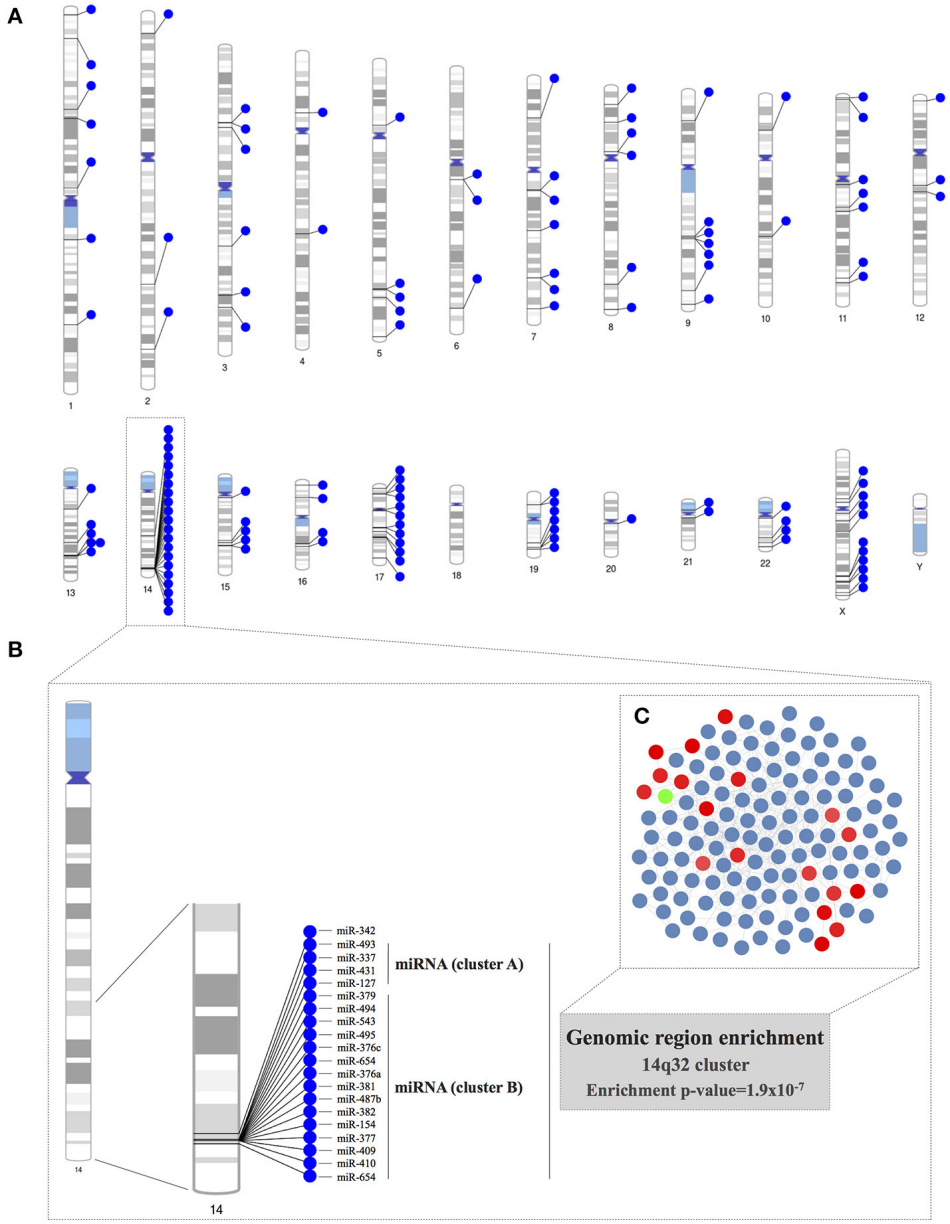
Genomic region enrichment analysis of the miRNA co-expression network. **(A)** The locus of each of the miRNAs included in the co-expression network was mapped in the chromosome ideograms to visualize the genomic loci of co-expressed miRNAs. **(B)** Detailed genomic localization of the co-expressed miRNAs located on chromosome 14. The genomic region overrepresentation was corroborated by the results of a hypergeometric test (*p* = 1.9 × 10^−7^). **(C)** The miRNAs located in the 14q32 locus are highlighted within the miRNA co-expression network.

We constructed chromosome ideograms to map the genomic locus of each of the miRNAs included in the co-expression network (Figure 3A). We found that 21 of the 131 PECAM^+^ EMP-associated co-expressed miRNAs were located on chromosome 14 and that 19 of them were located in the same chromosome region, the 14q32 locus. We confirmed the statistical significance of this 14q32 genomic region overrepresentation using a hypergeometric test (*p* = 1.90 × 10^−7^). This genomic location consists of the miRNA clusters A and B and is also known as the *Dlk1*-*Dio3* miRNA cluster. Specifically, we identified 4 of the 8 miRNAs located within miRNA cluster A (miR-127-3p, miR-337-3p, miR-431-5p, and miR-493-5p) and 15 of the 42 miRNAs located within miRNA cluster B (miR-154-5p, miR-376a-3p, miR-376c-3p, miR-377-3p, miR-379-5p, miR-381-3p, miR-382-5p, miR-409-3p, miR-410, miR-487b, miR-494, miR-495-3p, miR-543, miR-654-3p, and miR-654-5p; Figures 3B–C).

### Functional analysis of the 14q32 miRNA cluster

**Figure 4:**
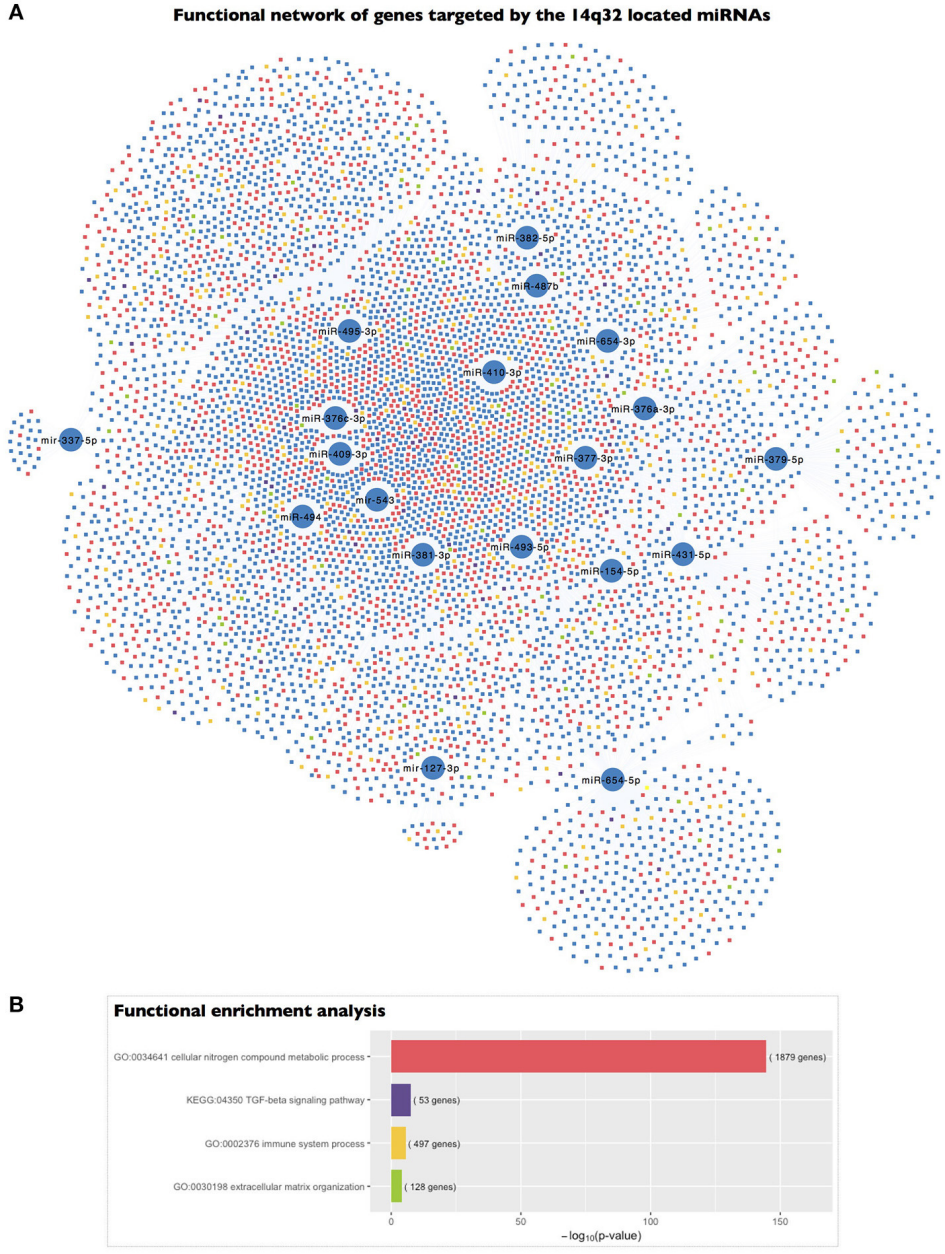
Functional enrichment analysis of the genes targeted by the 14q32 miRNAs represented in the miRNA co-expression analysis. **(A)** A network representing the genes targeted by each of the miRNAs included in the miRNA co-expression network and located within the 14q32 locus and their association with the highlighted biological process gene ontologies. **(B)** The significance of the functional enrichment analysis of the genes targeted by the 14q32-located miRNAs.

To gain deeper insight into the biological significance of these findings, we determined the biological significance of the regulation of the 14q32 miRNA cluster based on the functional implications of the genes targeted by the selected miRNAs. We carried out a functional enrichment analysis of the set of genes putatively targeted by the 19 EMP-related miRNAs located in the 14q32 cluster. Interestingly, this functional analysis revealed that the genes targeted by the 14q32 co-expressed miRNAs were categorized with the “cellular nitrogen compound metabolic process” (*p* = 2.34 × 10^−145^), “immune system process” (*p* = 2.57 × 10^−6^), and “extracellular matrix organization” (*p* = 8.14 × 10^−5^) GO terms as well as the “TGF-β signaling pathway” (*p* = 2.59 × 10^−8^) KEGG term, among others (Figure 4).

## Discussion

Using a bioinformatics approach, our study reported that the endothelial damage observed in BAV disease results in the differential regulation of a post-transcriptional regulatory miRNA network associated with the increased release of endothelial-derived microparticles. By determining the circulating miRNAs associated with PECAM^+^ EMP levels, we inferred a miRNA co-expression network. Furthermore, based on the results of genomic enrichment analysis, we focused on a cluster of highly co-expressed miRNAs located at the 14q32 locus on chromosome 14 that could be acting as molecular effectors in BAV-related pathophysiological processes, including endothelial damage. We showed that the miRNAs contained within the 14q32 miRNA cluster could mediate crucial biological processes in BAV disease, such as nitric oxide biosynthesis, immune activation, reorganization of the extracellular matrix and TGF-β signaling.

Bicuspid morphology of the aortic valve is associated with haemodynamic abnormalities that result in increased wall shear stress on the endothelial layer, which contributes to dilation of the ascending aorta (Braverman et al., 2005). At the cellular level, this disturbed blood flow is associated with rearrangement of the extracellular matrix and with endothelial damage and dysfunction (Davignon and Ganz, 2004). Furthermore, in our previous studies, we demonstrated that BAV and dilation of the aorta are associated with endothelial-mediated release of microparticles (Alegret et al., 2016). Thereby, when designing this study, we hypothesized that the characteristics of blood flow through the ascending aorta in patients with BAV disease are related to aortic endothelial damage and play key a role in EMP generation.

It is important to consider that on the one hand, circulating miRNAs take very stable forms, mainly packaged in transport particles; circulating microparticles have been reported as the major carriers of miRNAs in the blood (Diehl et al., 2012). On the other hand, although most published studies have focused mainly on differences in the expression of single miRNAs instead of studying miRNA co-expression networks, miRNAs are significantly enriched in clusters in discrete genomic regions (Lau, 2001; Kim and Nam, 2006), and miRNAs in the same cluster might be transcribed in a polycistronic manner (Baskerville, 2005; Wang et al., 2016) and likely regulate functionally related genes (Kim et al., 2009; Wang et al., 2011). We considered the determination and analysis of the miRNA co-expression network resulting from the integration of PECAM^+^ EMP levels with the expression of circulating miRNAs as an advantageous strategy to unravel the molecular mechanisms underlying the pathophysiological processes in BAV disease for the following reasons: changes in haemodynamic forces in the vascular system might alter the expression of miRNAs in endothelial cells (Marin et al., 2013); BAV promotes endothelial dysfunction, which results in the release of PECAM^+^ EMPs in plasma; microparticles are the main carriers of miRNAs in plasma; and miRNA-coding genes are enriched in discrete genomic regions.

Using a bioinformatics approach, we identified 175 miRNAs that were significantly associated with PECAM^+^ EMP levels. Of these miRNAs, 131 exhibited significant pairwise partial correlations and were inferred into a co-expression network to analyse miRNA co-expression patterns. Co-expression network analysis is considered a powerful method to extract strong regulatory associations that could be responsible for modulating transcriptional networks underlying biological processes. Accordingly, we first analyzed previous knowledge regarding the biological implications of the inferred network and found that 75% of the co-expressed miRNAs had been previously described as miRNAs expressed in endothelial cells and that 67% of them had been associated with cardiovascular diseases. We also analyzed the topology of the co-expression network and found that miR-494 was the most important hub of the network because it was the most highly connected miRNA. miR-494 was also the most influential miRNA within the network based on centrality parameters, which showed that miR-494 was most often found on the shortest path between two other miRNAs. These results indicate that miR-494 might have a large regulatory impact on the biological functions that could be modulated by the co-expression network.

We further analyzed the miRNA co-expression network in terms of enrichment for specific genomic locations, and we found that the 14q32 locus on chromosome 14 was significantly overrepresented as a highly co-expressed miRNA cluster for the PECAM^+^ EMP-associated and co-expressed miRNAs in BAV. This genomic region contains the largest miRNA cluster found in the human genome (Benetatos et al., 2013). Specifically, we found that 19 of the 131 miRNAs inferred in the co-expression network, including miR-494, are located within this chromosome region. This 14q32 miRNA cluster is also known as the imprinted *DLK1-MEG3* genomic region. This region contains the protein-coding genes *DLK1, RTL1*, and *DIO3*, which are expressed from the paternally inherited chromosome. The region also contains multiple long and short non-protein coding RNAs, including *MEG3, MEG8*, the miRNA cluster and small nucleolar RNA (snoRNA) genes, which are expressed from the maternally inherited chromosome. The imprinting of a genomic region refers to biased expression of the genes contained in either the paternally or maternally inherited chromosome instead of the more common biallelic expression. Although, miRNAs located within the 14q32 region have been proposed as candidates for various diseases, including cancer, psychiatric illness, alcoholism, and non-alcoholic fatty liver disease, to date, there is no published data relating the 14q32 miRNA cluster with cardiovascular diseases (Benetatos et al., 2013; Okamoto et al., 2016).

Based on the functional enrichment of the genes targeted by the PECAM^+^ EMP-associated and co-expressed miRNAs located within the 14q32 locus, we could determine the biological significance of the regulation of this miRNA cluster. We found that the genes targeted by the 14q32 co-expressed miRNAs are strongly involved in the regulation of genes in categories such as “cellular nitrogen compound metabolic process,” “immune system process,” “extracellular matrix organization,” and “TGF-β signaling pathway.” The “cellular nitrogen compound metabolic process” gene ontology term includes the “nitric oxide biosynthetic pathway” subcategory. Nitric oxide (NO) is a pivotal endothelium-derived substance that plays a crucial role in the homeostasis of the cardiovascular system by modulating endothelium-dependent vasodilation; in fact, impairment of NO production or activity has been proposed as a major mechanism of endothelial dysfunction (Davignon and Ganz, 2004). Moreover, modulation of the expression and activity of endothelial nitric oxide synthase (eNOS), the specific enzyme that produces NO in endothelial cells, by fluid shear stress and the implication of this modulation in the development of BAV are well established (Ranjan et al., 1995; Aicher et al., 2007; Vion et al., 2013). Thus, in the context of BAV, the valvulopathy and the associated abnormal haemodynamics are related to the altered distribution of aortic wall shear stress, which promotes regional disruption of the eNOS pathway in a manner that is primarily mediated by the differential expression and activity of eNOS. Endothelial cells release EMPs in response to cellular stress and cell activation (Dignat-George and Boulanger, 2011). Consistent with this fact and the role of PECAM^+^ EMP-related and co-expressed miRNAs in the regulation of endothelial dysfunction, we found that among the genes targeted by the 14q32-located miRNAs, those with functional implications related to modulation of immune system processes and to extracellular matrix organization were significantly enriched. In fact, interactions between inflammatory activation and endothelial dysfunction have been previously described in patients with BAV (Ali et al., 2014). In addition, we also found that the 14q32-located miRNAs present in our co-expression network targeted genes that were related to the TGF-β signaling pathway. The TGF-β family is composed of several cytokines with diverse functions, including the regulation of tissue repair and fibrosis, extracellular matrix remodeling, and inflammation as well as cell proliferation and migration (Bobik, 2006). Previous studies have implicated the impairment of TGF-β signaling in BAV pathology and the development of BAV-associated aortopathy (Forte et al., 2016). Additionally, functional interactions between TGF-β and NO have been demonstrated in endothelial cells (Saura et al., 2005). All these data suggest that the 14q32 miRNA cluster may play a pivotal role in the induction of BAV-associated endothelial damage.

Furthermore, in addition to studying the post-transcriptional implications of the regulatory functions of the 14q32 miRNA cluster, we thought it would be interesting to understand the regulatory mechanisms that could affect the activation or repression of the expression of this miRNA cluster. The imprinted status of the maternally expressed RNAs of the *DLK1-MEG3* locus, including the 14q32 miRNA cluster, is regulated by epigenetic mechanisms, specifically by the methylation of two differentially methylated regions (DMRs) located upstream of the transcription activation site of *MEG3* (Murphy et al., 2003; Kameswaran et al., 2014). The hypermethylation of either of these two DMRs has been associated with decreased expression of the maternal transcript (Kagami et al., 2010). In various models, such as in human type 2 diabetic islets, repression of the 14q32 miRNA cluster is strongly correlated with hypermethylation of the *MEG3*-DMR, and modifications at this region increase susceptibility to disease (Kameswaran et al., 2014). Interestingly, Boon et al. (2016) proposed that aging induces the expression of *MEG3* and that *MEG3*-mediated changes in the epigenetic regulation of gene expression contributes to aging-related endothelial dysfunction. Moreover, Gordon et al. (2010) suggested that *MEG3* may regulate the expression of *VEGF*; they showed that loss of *MEG3* leads to up-regulation of the expression of genes in the VEGF and Notch signaling pathways in mouse brains. Furthermore, DNA methylation mechanisms are responsive to disturbed flow (Dunn et al., 2014); in fact, endothelial gene expression can be regulated by flow-dependent epigenetic mechanisms (Jiang et al., 2015). Therefore, the disturbed flow associated with BAV may promote not only the secretion of EMPs but also the *MEG3*-mediated epigenetic regulation of the 14q32 miRNA cluster and thus may play a key role in the regulation of endothelial damage in BAV disease. On the other hand, DLK1 is the best-studied non-canonical Notch ligand and acts as an inhibitor of Notch signaling *in vitro* (Baladrón et al., 2005). In this manner, regulation of the expression of genes located in the 14q32 locus could also modulate the crosstalk between TGF-β and the VEGF and Notch signaling pathways, which have been previously described to act co-ordinately in space and time in the regulation of vascular morphogenesis (Holderfield and Hughes, 2008).

## Limitations

We studied the miRNAs associated with the secretion of PECAM^+^ EMPs in the context of BAV-induced endothelial damage, but we did not determine if this pattern of expression of PECAM^+^ EMPs-associated miRNAs is also deregulated when the endothelial damage is caused by other conditions.

In summary, we propose that the changes in hemodynamic forces in the vascular system that are caused by the bicuspid morphology of the aortic valve might result in the expression of a specific pattern of PECAM^+^ EMP-associated miRNAs that regulates a potent post-transcriptional network that might be involved in the regulation of endothelial damage. We reported that the highly co-expressed and PECAM^+^ EMP-associated miRNAs clustered within the 14q32 locus might modulate a functional regulatory miRNA co-expression network that targets functionally related genes, which could be the molecular effectors linking the impairment of various signaling pathways (VEGFA, TGF-β, NOTCH) involved in the pathophysiology of BAV disease. Thus, we propose that the 14q32 miRNAs may act as master switches for endothelial damage and that inhibition of either individual or a combination of 14q32 miRNAs might offer a new therapeutic approach for BAV disease.

## Author contributions

NM: Performed the experiments, analyzed the data, and wrote the manuscript; RB and GA: Performed the experiments; MF: Contributed in sample collection and writing the manuscript; JA: Conceived and designed the study, analyzed the data and revised the manuscript. All authors read and approved the final manuscript.

### Conflict of interest statement

The authors declare that the research was conducted in the absence of any commercial or financial relationships that could be construed as a potential conflict of interest.

## References

[B1] Agarwal V. Bell G. W. Nam J.-W. Bartel D. P. (2015). Predicting effective microRNA target sites in mammalian mRNAs. Elife 4:e05005. 10.7554/eLife.05005 26267216 PMC4532895

[B2] Aicher D. Urbich C. Zeiher A. Dimmeler S. Schäfers H.-J. (2007). Endothelial nitric oxide synthase in bicuspid aortic valve disease. Ann. Thorac. Surg. 83, 1290–1294. 10.1016/j.athoracsur.2006.11.086 17383329

[B3] Alegret J. M. Ligero C. Vernis J. M. Beltrán-Debón R. Aragonés G. Duran I. (2013). Factors related to the need for surgery after the diagnosis of bicuspid aortic valve: one center's experience under a conservative approach. Int. J. Med. Sci. 10, 176–182. 10.7150/ijms.5399 23329890 PMC3547216

[B4] Alegret J. M. Martínez-Micaelo N. Aragonès G. Beltrán-Debón R. (2016). Circulating endothelial microparticles are elevated in bicuspid aortic valve disease and related to aortic dilation. Int. J. Cardiol. 217, 35–41. 10.1016/j.ijcard.2016.04.184 27179206

[B5] Alegret J. M. Palazón O. Duran I. Vernis J. M. (2005). Aortic valve morphology definition with transthoracic combined with transesophageal echocardiography in a population with high prevalence of bicuspid aortic valve. Int. J. Cardiovasc. Imaging 21, 213–217. 10.1007/s10554-004-3901-9 16015430

[B6] Ali O. A. Chapman M. Nguyen T. H. Chirkov Y. Y. Heresztyn T. Mundisugih J.. (2014). Interactions between inflammatory activation and endothelial dysfunction selectively modulate valve disease progression in patients with bicuspid aortic valve. Heart 100, 800–805. 10.1136/heartjnl-2014-305509 24743038

[B7] Amabile N. Guérin A. P. Leroyer A. Mallat Z. Nguyen C. Boddaert J.. (2005). Circulating endothelial microparticles are associated with vascular dysfunction in patients with end-stage renal failure. J. Am. Soc. Nephrol. 16, 3381–3388. 10.1681/ASN.2005050535 16192427

[B8] Baladrón V. Ruiz-Hidalgo M. J. Nueda M. L. Díaz-Guerra M. J. M. García-Ramírez J. J. Bonvini E.. (2005). dlk acts as a negative regulator of Notch1 activation through interactions with specific EGF-like repeats. Exp. Cell Res. 303, 343–359. 10.1016/j.yexcr.2004.10.001 15652348

[B9] Baskerville S. (2005). Microarray profiling of microRNAs reveals frequent coexpression with neighboring miRNAs and host genes. RNA 11, 241–247. 10.1261/rna.7240905 15701730 PMC1370713

[B10] Benetatos L. Hatzimichael E. Londin E. Vartholomatos G. Loher P. Rigoutsos I.. (2013). The microRNAs within the DLK1-DIO3 genomic region: involvement in disease pathogenesis. Cell. Mol. Life Sci. 70, 795–814. 10.1007/s00018-012-1080-8 22825660 PMC11114045

[B11] Benjamini Y. Hochberg Y. (1995). Controlling the false discovery rate: a practical and powerful approach to multiple testing. J. R. Stat. Soc. Ser. B 57, 289–300.

[B12] Biner S. Rafique A. M. Ray I. Cuk O. Siegel R. J. Tolstrup K. (2009). Aortopathy is prevalent in relatives of bicuspid aortic valve patients. J. Am. Coll. Cardiol. 53, 2288–2295. 10.1016/j.jacc.2009.03.027 19520254 PMC2761956

[B13] Bissell M. M. Hess A. T. Biasiolli L. Glaze S. J. Loudon M. Pitcher A.. (2013). Aortic dilation in bicuspid aortic valve disease: flow pattern is a major contributor and differs with valve fusion type. Circ. Cardiovasc. Imaging 6, 499–507. 10.1161/CIRCIMAGING.113.000528 23771987 PMC3859916

[B14] Bobik A. (2006). Transforming growth factor-betas and vascular disorders. Arterioscler. Thromb. Vasc. Biol. 26, 1712–1720. 10.1161/01.ATV.0000225287.20034.2c 16675726

[B15] Boon R. A. Hofmann P. Michalik K. M. Lozano-Vidal N. Berghäuser D. Fischer A.. (2016). Long noncoding RNA Meg3 controls endothelial cell aging and function. J. Am. Coll. Cardiol. 68, 2589–2591. 10.1016/j.jacc.2016.09.949 27931619

[B16] Braverman A. C. Güven H. Beardslee M. A. Makan M. Kates A. M. Moon M. R.. (2005). The bicuspid aortic valve. Curr. Probl. Cardiol. 30, 470–522. 10.1016/j.cpcardiol.2005.06.002 16129122

[B17] Ci H.-B. Ou Z.-J. Chang F.-J. Liu D.-H. He G.-W. Xu Z.. (2013). Endothelial microparticles increase in mitral valve disease and impair mitral valve endothelial function. Am. J. Physiol. Endocrinol. Metab. 304, E695–E702. 10.1152/ajpendo.00016.2013 23384770

[B18] Cline M. S. Smoot M. Cerami E. Kuchinsky A. Landys N. Workman C.. (2007). Integration of biological networks and gene expression data using cytoscape. Nat. Protoc. 2, 2366–2382. 10.1038/nprot.2007.324 17947979 PMC3685583

[B19] Davignon J. Ganz P. (2004). Role of endothelial dysfunction in atherosclerosis. Circulation 109, III27–III32. 10.1161/01.CIR.0000131515.03336.f8 15198963

[B20] Diehl P. Fricke A. Sander L. Stamm J. Bassler N. Htun N.. (2012). Microparticles: major transport vehicles for distinct microRNAs in circulation. Cardiovasc. Res. 93, 633–644. 10.1093/cvr/cvs007 22258631 PMC3291092

[B21] Dignat-George F. Boulanger C. M. (2011). The many faces of endothelial microparticles. Arterioscler. Thromb. Vasc. Biol. 31, 27–33. 10.1161/ATVBAHA.110.218123 21160065

[B22] Dunn J. Qiu H. Kim S. Jjingo D. Hoffman R. Kim C. W.. (2014). Flow-dependent epigenetic DNA methylation regulates endothelial gene expression and atherosclerosis. J. Clin. Invest. 124, 3187–3199. 10.1172/JCI74792 24865430 PMC4071393

[B23] Forte A. Galderisi U. Cipollaro M. De Feo M. Corte A. D. (2016). Epigenetic regulation of TGF- 1 signalling in dilative aortopathy of the thoracic ascending aorta. Clin. Sci. 130, 1389–1405. 10.1042/CS20160222 27389586

[B24] Gleeson T. G. Mwangi I. Horgan S. J. Cradock A. Fitzpatrick P. Murray J. G. (2008). Steady-state free-precession (SSFP) cine MRI in distinguishing normal and bicuspid aortic valves. J. Magn. Reson. Imaging 28, 873–878. 10.1002/jmri.21547 18821622

[B25] Gordon F. E. Nutt C. L. Cheunsuchon P. Nakayama Y. Provencher K. A. Rice K. A.. (2010). Increased expression of angiogenic genes in the brains of mouse meg3-null embryos. Endocrinology 151, 2443–2452. 10.1210/en.2009-1151 20392836 PMC2875815

[B26] Holderfield M. T. Hughes C. C. W. (2008). Crosstalk between vascular endothelial growth factor, notch, and transforming growth factor-beta in vascular morphogenesis. Circ. Res. 102, 637–652. 10.1161/CIRCRESAHA.107.167171 18369162

[B27] Irizarry R. A. Hobbs B. Collin F. Beazer-Barclay Y. D. Antonellis K. J. Scherf U.. (2003). Exploration, normalization, and summaries of high density oligonucleotide array probe level data. Biostatistics 4, 249–264. 10.1093/biostatistics/4.2.249 12925520

[B28] Jiang Y.-Z. Manduchi E. Stoeckert C. J. Davies P. F. (2015). Arterial endothelial methylome: differential DNA methylation in athero-susceptible disturbed flow regions *in vivo*. BMC Genomics 16:506. 10.1186/s12864-015-1656-4 26148682 PMC4492093

[B29] Jimenez J. J. Jy W. Mauro L. M. Soderland C. Horstman L. L. Ahn Y. S. (2003). Endothelial cells release phenotypically and quantitatively distinct microparticles in activation and apoptosis. Thromb. Res. 109, 175–180. 10.1016/S0049-3848(03)00064-1 12757771

[B30] Kagami M. O'Sullivan M. J. Green A. J. Watabe Y. Arisaka O. Masawa N.. (2010). The IG-DMR and the MEG3-DMR at Human Chromosome 14q32.2: Hierarchical interaction and distinct functional properties as imprinting control centers. PLoS Genet. 6:e1000992. 10.1371/journal.pgen.1000992 20585555 PMC2887472

[B31] Kameswaran V. Bramswig N. C. McKenna L. B. Penn M. Schug J. Hand N. J.. (2014). Epigenetic regulation of the DLK1-MEG3 microRNA cluster in human type 2 diabetic islets. Cell Metab. 19, 135–145. 24374217 10.1016/j.cmet.2013.11.016 PMC3932527

[B32] Kim V. N. Nam J.-W. (2006). Genomics of microRNA. Trends Genet. 22, 165–173. 10.1016/j.tig.2006.01.003 16446010

[B33] Kim Y.-G. Sun B. J. Park G.-M. Han S. Kim D.-H. Song J.-M.. (2012). Aortopathy and bicuspid aortic valve: haemodynamic burden is main contributor to aortic dilatation. Heart 98, 1822–1827. 10.1136/heartjnl-2012-302828 23204534

[B34] Kim Y.-K. Yu J. Han T. S. Park S.-Y. Namkoong B. Kim D. H.. (2009). Functional links between clustered microRNAs: suppression of cell-cycle inhibitors by microRNA clusters in gastric cancer. Nucleic Acids Res. 37, 1672–1681. 10.1093/nar/gkp002 19153141 PMC2655672

[B35] Lau N. C. (2001). An abundant class of tiny RNAs with probable regulatory roles in caenorhabditis elegans. Science 294, 858–862. 10.1126/science.1065062 11679671

[B36] Marin T. Gongol B. Chen Z. Woo B. Subramaniam S. Chien S.. (2013). Mechanosensitive microRNAs—role in endothelial responses to shear stress and redox state. Free Radic. Biol. Med. 64, 61–68. 10.1016/j.freeradbiomed.2013.05.034 23727269 PMC3762952

[B37] Martínez-Micaelo N. Beltrán-Debón R. Baiges I. Faiges M. Alegret J. M. (2017). Specific circulating microRNA signature of bicuspid aortic valve disease. J. Transl. Med. 15:76. 10.1186/s12967-017-1176-x 28399937 PMC5387230

[B38] Mourelatos Z. Dostie J. Paushkin S. Sharma A. Charroux B. Abel L.. (2002). miRNPs: a novel class of ribonucleoproteins containing numerous microRNAs. Genes Dev. 16, 720–728. 10.1101/gad.974702 11914277 PMC155365

[B39] Murphy S. K. Wylie A. A. Coveler K. J. Cotter P. D. Papenhausen P. R. Sutton V. R.. (2003). Epigenetic detection of human chromosome 14 uniparental disomy. Hum. Mutat. 22, 92–97. 10.1002/humu.10237 12815599

[B40] Okamoto K. Koda M. Okamoto T. Onoyama T. Miyoshi K. Kishina M.. (2016). A series of microRNA in the Chromosome 14q32.2 Maternally imprinted region related to progression of non-alcoholic fatty liver disease in a mouse model. PLoS ONE 11:e0154676. 10.1371/journal.pone.0154676 27135827 PMC4852931

[B41] Opgen-Rhein R. Strimmer K. (2007). From correlation to causation networks: a simple approximate learning algorithm and its application to high-dimensional plant gene expression data. BMC Syst. Biol. 1:37. 10.1186/1752-0509-1-37 17683609 PMC1995222

[B42] Padang R. Bannon P. G. Jeremy R. Richmond D. R. Semsarian C. Vallely M.. (2013). The genetic and molecular basis of bicuspid aortic valve associated thoracic aortopathy: a link to phenotype heterogeneity. Ann. Cardiothorac. Surg. 2, 83–91. 10.3978/j.issn.2225-319X.2012.11.17 23977563 PMC3741809

[B43] Paraskevopoulou M. D. Georgakilas G. Kostoulas N. Vlachos I. S. Vergoulis T. Reczko M.. (2013). DIANA-microT web server v5.0: service integration into miRNA functional analysis workflows. Nucleic Acids Res. 41, W169–W173. 10.1093/nar/gkt393 23680784 PMC3692048

[B44] Pepe G. Nistri S. Giusti B. Sticchi E. Attanasio M. Porciani C.. (2014). Identification of fibrillin 1 gene mutations in patients with bicuspid aortic valve (BAV) without Marfan syndrome. BMC Med. Genet. 15:23. 10.1186/1471-2350-15-23 24564502 PMC3937520

[B45] Pirro M. Schillaci G. Paltriccia R. Bagaglia F. Menecali C. Mannarino M. R.. (2006). Increased ratio of CD31+/CD42- microparticles to endothelial progenitors as a novel marker of atherosclerosis in hypercholesterolemia. Arterioscler. Thromb. Vasc. Biol. 26, 2530–2535. 10.1161/01.ATV.0000243941.72375.15 16946129

[B46] Ranjan V. Xiao Z. Diamond S. L. (1995). Constitutive NOS expression in cultured endothelial cells is elevated by fluid shear stress. Am. J. Physiol. 269, H550–H555. 7544542 10.1152/ajpheart.1995.269.2.H550

[B47] Ritchie M. E. Phipson B. Wu D. Hu Y. Law C. W. Shi W.. (2015). Limma powers differential expression analyses for RNA-sequencing and microarray studies. Nucleic Acids Res. 43:e47. 10.1093/nar/gkv007 25605792 PMC4402510

[B48] Saura M. Zaragoza C. Herranz B. Griera M. Diez-Marqués L. Rodriguez-Puyol D.. (2005). Nitric oxide regulates transforming growth factor-β signaling in endothelial cells. Circ. Res. 97, 115–123. 10.1161/01.RES.0000191538.76771.66 16239590

[B49] Schäfer J. Strimmer K. (2005). An empirical bayes approach to inferring large-scale gene association networks. Bioinformatics 21, 754–764. 10.1093/bioinformatics/bti062 15479708

[B50] Sutherland W. H. F. de Jong S. A. Hessian P. A. Williams M. J. A. (2010). Ingestion of native and thermally oxidized polyunsaturated fats acutely increases circulating numbers of endothelial microparticles. Metab. Clin. Exp. 59, 446–453. 10.1016/j.metabol.2009.07.033 19846183

[B51] Tzemos N. Therrien J. Yip J. Thanassoulis G. Tremblay S. Jamorski M. T.. (2008). Outcomes in adults with bicuspid aortic valves. JAMA 300, 1317–1325. 10.1001/jama.300.11.1317 18799444

[B52] Vion A.-C. Ramkhelawon B. Loyer X. Chironi G. Devue C. Loirand G.. (2013). Shear stress regulates endothelial microparticle release. Circ. Res. 112, 1323–1333. 10.1161/CIRCRESAHA.112.300818 23536307

[B53] Vlachos I. S. Paraskevopoulou M. D. Karagkouni D. Georgakilas G. Vergoulis T. Kanellos I.. (2015a). DIANA-TarBase v7.0: indexing more than half a million experimentally supported miRNA:mRNA interactions. Nucleic Acids Res. 43, D153–D159. 10.1093/nar/gku1215 25416803 PMC4383989

[B54] Vlachos I. S. Zagganas K. Paraskevopoulou M. D. Georgakilas G. Karagkouni D. Vergoulis T.. (2015b). DIANA-miRPath v3.0: deciphering microRNA function with experimental support. Nucleic Acids Res. 43, W460–W466. 10.1093/nar/gkv403 25977294 PMC4489228

[B55] Wang J. Haubrock M. Cao K.-M. Hua X. Zhang C.-Y. Wingender E.. (2011). Regulatory coordination of clustered microRNAs based on microRNA-transcription factor regulatory network. BMC Syst. Biol. 5:199. 10.1186/1752-0509-5-199 22176772 PMC3262773

[B56] Wang Y. Luo J. Zhang H. Lu J. (2016). microRNAs in the Same clusters evolve to coordinately regulate functionally related genes. Mol. Biol. Evol. 33, 2232–2247. 10.1093/molbev/msw089 27189568 PMC4989102

[B57] Wolfe D. Dudek S. Ritchie M. D. Pendergrass S. A. Ramos P. Criswell L.. (2013). Visualizing genomic information across chromosomes with PhenoGram. BioData Min. 6:18. 10.1186/1756-0381-6-18 24131735 PMC4015356

