# Supplementary material for: MicroRNAs Clustered within the 14q32 Locus Are Associated with Endothelial Damage and Microparticle Secretion in Bicuspid Aortic Valve Disease
Source: Front Physiol. 2017 Sep 5;8:648. doi: 10.3389/fphys.2017.00648 (PMC5591958; doi:10.3389/fphys.2017.00648)

MicroRNAs clustered within the 14q32 locus are associated with endothelial damage and microparticle secretion in bicuspid aortic valve disease

Neus Martínez-Micaelo[1*], Raúl Beltrán-Debón[1], Gerard Aragonés[1], Marta Faiges[1], Josep M. Alegret[1, 2*]

[1]Grup de Recerca Cardiovascular, Institut d'Investigació Sanitària Pere Virgili (IISPV); Universitat Rovira i Virgili, Reus, Spain.

[2] Servei de Cardiologia, Hospital Universitari de Sant Joan, Universitat Rovira i Virgili, Reus, Spain.

Running title: EMP-associated miRNAs and endothelial damage

Keywords: microRNA, bicuspid aortic valve, aortic dilation

Correspondings authors:

Josep M Alegret and Neus Martínez-Micaelo

Grup de Recerca Cardiovascular, IISPV

Servei de Cardiologia, Hospital Universitari de Sant Joan

Departament de Medicina i Cirurgia, Universitat Rovira i Virgili

c/ Dr Josep Laporte, 1

43204 Reus;

Phone: +34 977310300; Fax: +34977315144

Email: josepmaria.alegret@urv.cat

neus.martinez@urv.cat

Supplementary Table 1. The clinical and echocardiographic characteristics of the patients included in the microarray analysis.

	**TAV**	**BAV**	**p-value**
Age (years)	41 ± 4	37 ± 2	0.405
Sex (male/female)	(6/0)	(18/0)	1.000
Body weight (kg)	74.2 ± 2.2	77.0 ± 3.5	0.496
Hypertension	0 (0%)	0 (0%)	1.000
Hypercholesterolemia	0 (0%)	2 (11.1%)	0.394
Smoker	1 (20.0%)	5 (27.8%)	0.726
Aortic stenosis (mean gradient ≥20 mm Hg)	0 (0%)	0 (0%)	1.00
Aortic regurgitation (≥II)	0 (0%)	9 (50.0%)	0.028*
Aortic valve gradient (mean, mm Hg)	3.75 ± 0.3	6.0 ± 01.0	0.036*
Left ventricle diastolic diameter (mm)	51.67 ± 1.5	53.06 ± 1.0	0.472
Left ventricle systolic diameter (mm)	32.33 ± 0.9	32.88 ± 0.9	0.734
Left ventricular ejection fraction (%)	74.40 ± 3.2	71.30 ± 1.4	0.340
Aortic root diameter (mm/m^2)	15.88 ± 0.6	19.15 ± 0.7	2.9×10^{-3}**
Ascending aorta diameter (mm/m^2)	15.05 ± 0.5	18.23 ± 0.9	5.5×10^{-3}**

* Significant values (p<0.05); ** Significant values (p<0.01); TAV, Tricuspid aortic valve patients; BAV, Bicuspid aortic valve patients.

Supplementary Table 2. The clinical and echocardiographic characteristics of the patients included in the validation study.

	**TAV**	**BAV**	**p-value**
Age (years)	38 ± 3	37 ± 2	0.643
Sex (male/female)	(8/4)	(17/7)	0.798
Body weight (kg)	70.1 ± 3.7	70.0 ± 3.1	0.973
Hypertension	0 (0%)	4 (16.7%)	0.134
Hypercholesterolemia	1 (8.3%)	0 (0%)	0.151
Smoker	0 (0%)	6 (25.0%)	0.190
Aortic stenosis (mean gradient ≥20 mm Hg)	0 (0%)	3 (12.5%)	0.201
Aortic regurgitation (≥II)	0 (0%)	11 (45.8%)	$4.8{\times}10^{-3}$**
Aortic valve gradient (mean, mm Hg)	3.6 ± 0.2	11.9 ± 1.5	$9.1{\times}10^{-3}$**
Left ventricle diastolic diameter (mm)	50.1 ± 1.2	52.2 ± 1.1	0.229
Left ventricle systolic diameter (mm)	29.9 ± 0.7	32.0 ± 0.8	0.099
Left ventricular ejection fraction (%)	77.3 ± 1.6	71.8 ± 4.8	$5.1{\times}10^{-3}$**
Aortic root diameter (mm/m^2)	16.6 ± 0.7	20.3 ± 0.6	$8.7{\times}10^{-3}$**
Ascending aorta diameter (mm/m^2)	15.9 ± 0.6	21.2 ± 1.2	$3.6{\times}10^{-4}$**

* Significant values (p<0.05); ** Significant values (p<0.01); TAV, Tricuspid aortic valve patients; BAV, Bicuspid aortic valve patients.

Supplementary Figure 1. The bicuspid morphology of the aortic valve and dilation of the aorta are associated with increased circulating levels of EMPs, which, in turn, are correlated with miRNA expression in plasma. (A) The levels of circulating EMPs were significantly higher in the BAV patients compared with the controls. (B) Dilation of the aortic root or the ascending aorta was significantly associated with increased levels of EMPs in plasma. (C) The results of the association between the miRNA expression and the EMP levels are presented. For each miRNA included in the set, its –log10-transformed p-value corresponding to the association between miRNA expression and EMP level is represented on the y-axis.

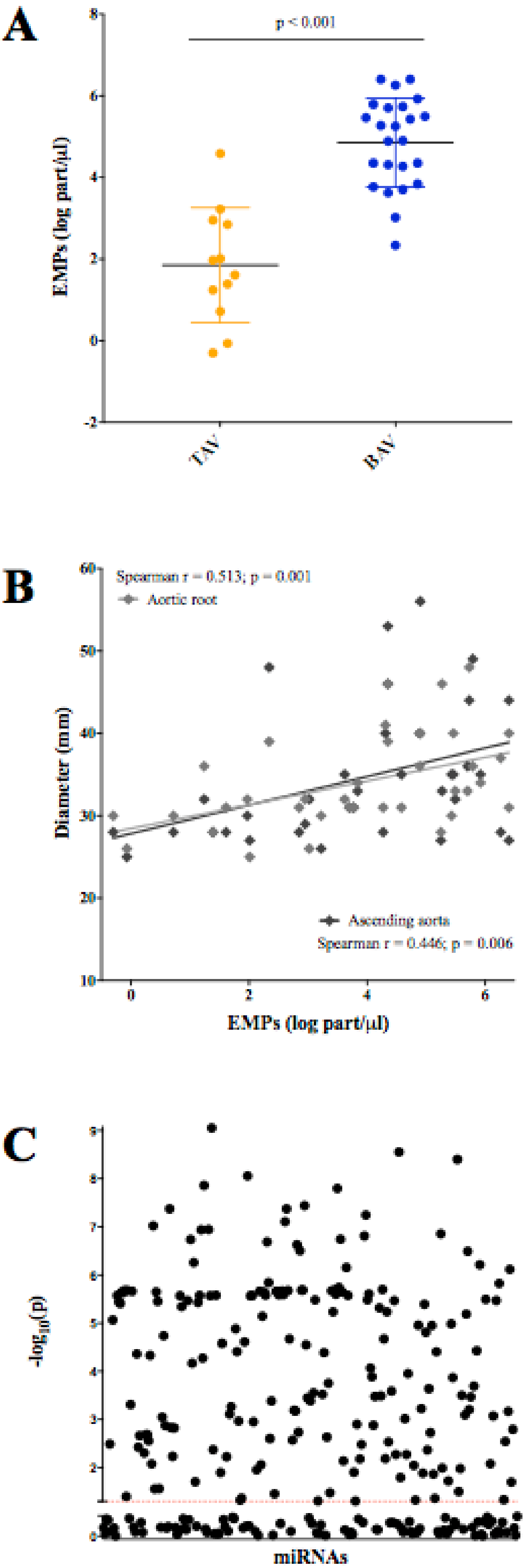

Supplement: Supplementary file 1 [file Table1.PDF]
